# Supplementary figures and images for: UDP-glucose pyrophosphorylase: genome-wide identification, expression and functional analyses in Gossypium hirsutum
Source: PeerJ. 2022 May 30;10:e13460. doi: 10.7717/peerj.13460 (PMC9161816; doi:10.7717/peerj.13460)

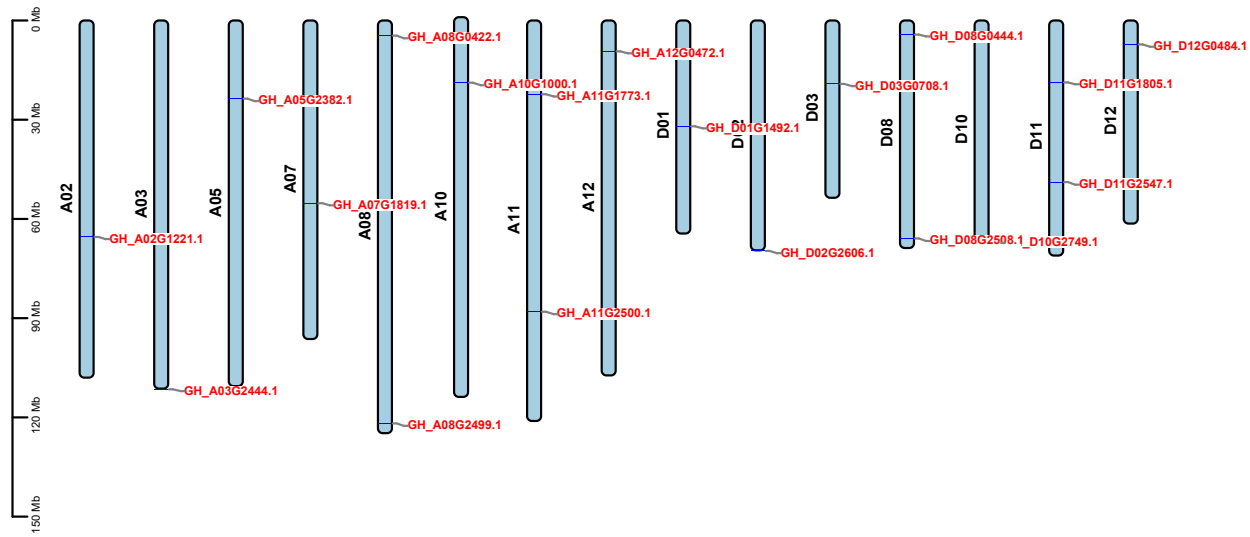

Supplement: Supplemental Information 1 [file peerj-10-13460-s001.pdf]
